# Supplementary figures and images for: Enhancing neural collaborative filtering using hybrid feature selection for recommendation
Source: PeerJ Comput Sci. 2023 Aug 28;9:e1456. doi: 10.7717/peerj-cs.1456 (PMC10496003; doi:10.7717/peerj-cs.1456)

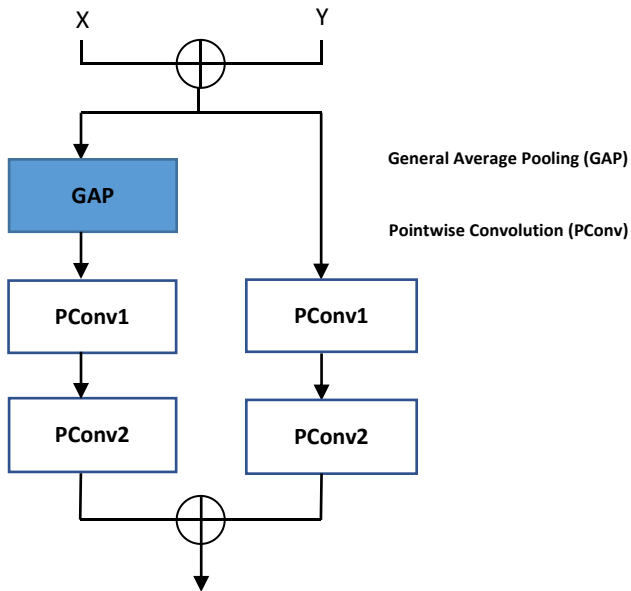

Supplement: Supplemental Information 1 [file peerj-cs-09-1456-s001.pdf]

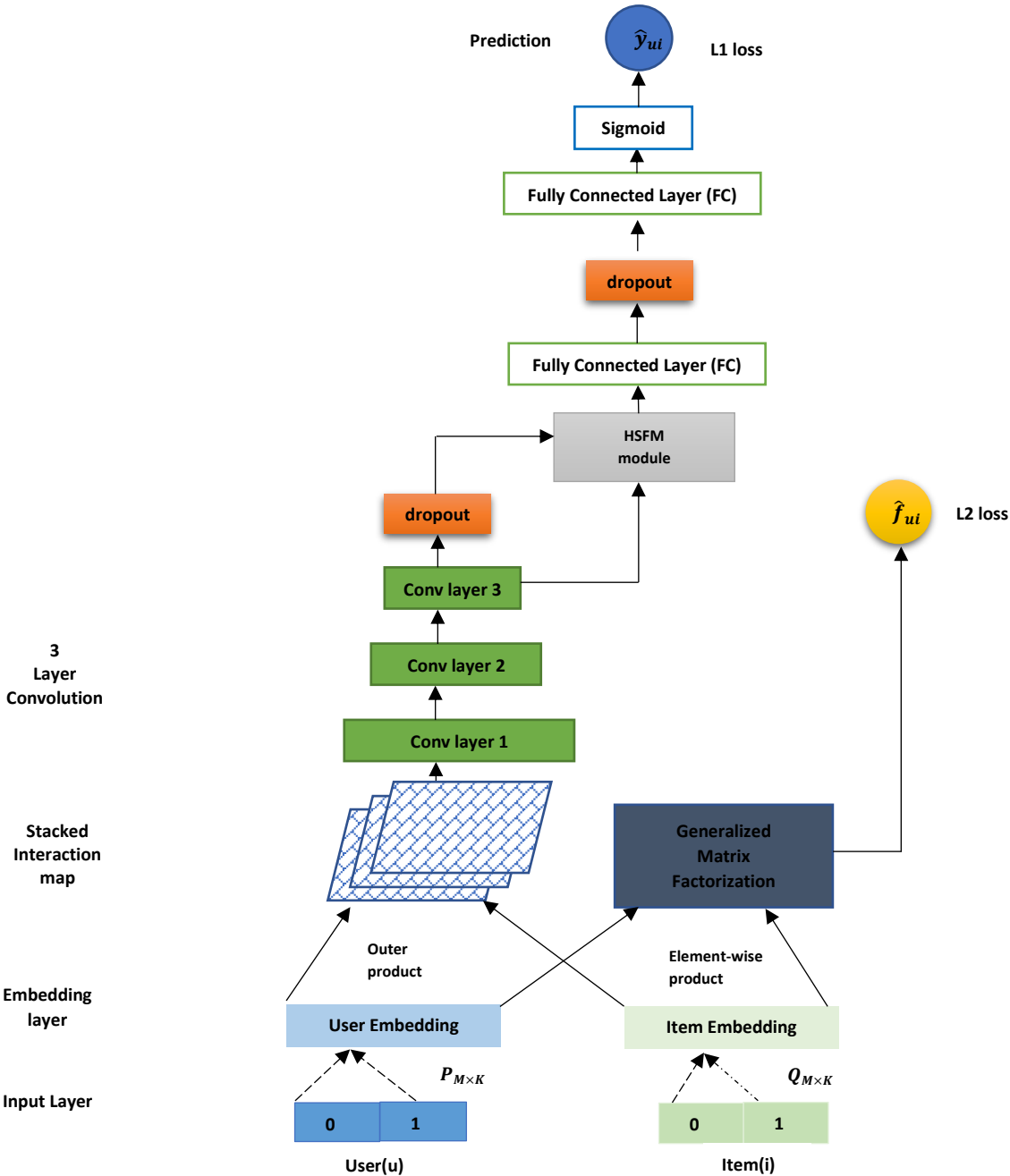

Supplement: Supplemental Information 2 [file peerj-cs-09-1456-s002.pdf]
