# Supplementary material for: Enhancing neural collaborative filtering using hybrid feature selection for recommendation
Source: PeerJ Comput Sci. 2023 Aug 28;9:e1456. doi: 10.7717/peerj-cs.1456 (PMC10496003; doi:10.7717/peerj-cs.1456)
Supplement: Supplemental Information 3 [file peerj-cs-09-1456-s003.docx]

The datasets used to evaluate the performance of our model are movielens and Pinterest. We preprocess the dataset using the same format as (He et al., 2017)

| Dataset | #Users | #Items | Interactions | Sparsity |
| --- | --- | --- | --- | --- |
| Ml-1M | 6040 | 3706 | 10000209 | 95.53% |
| Pinterest | 55187 | 9916 | 1500809 | 99.73% |
